# Molecules and mechanisms controlling the active DNA demethylation of the mammalian zygotic genome

**DOI:** 10.1007/s13238-014-0095-3

**Published:** 2014-08-26

**Authors:** Jun-Yu Ma, Teng Zhang, Wei Shen, Heide Schatten, Qing Yuan Sun

**Affiliations:** 1 College of Animal Science and Technology, Qingdao Agricultural University, Qingdao, 266109 China; 2 State Key Laboratory of Reproductive Biology, Institute of Zoology, Chinese Academy of Sciences, Beijing, 100101 China; 3 Department of Veterinary Pathobiology, University of Missouri, Columbia, MO 65211 USA

**Keywords:** active DNA demethylation, zygote, 5-hmC, 5-mC, preimplantation embryo, TET proteins

## Abstract

The active DNA demethylation in early embryos is essential for subsequent development. Although the zygotic genome is globally demethylated, the DNA methylation of imprinted regions, part of repeat sequences and some gamete-specific regions are maintained. Recent evidence has shown that multiple proteins and biological pathways participate in the regulation of active DNA demethylation, such as TET proteins, DNA repair pathways and DNA methyltransferases. Here we review the recent understanding regarding proteins associated with active DNA demethylation and the regulatory networks controlling the active DNA demethylation in early embryos.

## Introduction

Prior to fertilization, both the oocyte and spermatozoon have formed a gamete-specific DNA methylation pattern and specific genomic imprinting (Kobayashi et al., 2012; Smith et al., 2012). For the spermatozoon, 80%–90% of low density CpG sites are methylated whereas only 50% are methylated in the oocyte (Kobayashi et al., 2012). For the moderate or high density CpG sites, both oocyte and sperm genomes are hypomethylated. At the transposable element DNA regions, the sperm genome is highly methylated but the oocyte genome is only moderately methylated (Kobayashi et al., 2012). Reduced representation bisulfite sequencing result showed that the number of oocyte-specific methylated DNA regions was 376 and that of the spermatozoon was 4894 (Smith et al., 2012). In addition to the imprinting control regions (ICRs) and gamete-specific methylated CpG sites, whole-genome shotgun bisulfite sequencing data of the oocyte and spermatozoon also showed that more than 1600 CpG islands (CGIs) were differentially methylated between oocytes and spermatozoa (Kobayashi et al., 2012).

**Figure 1:**
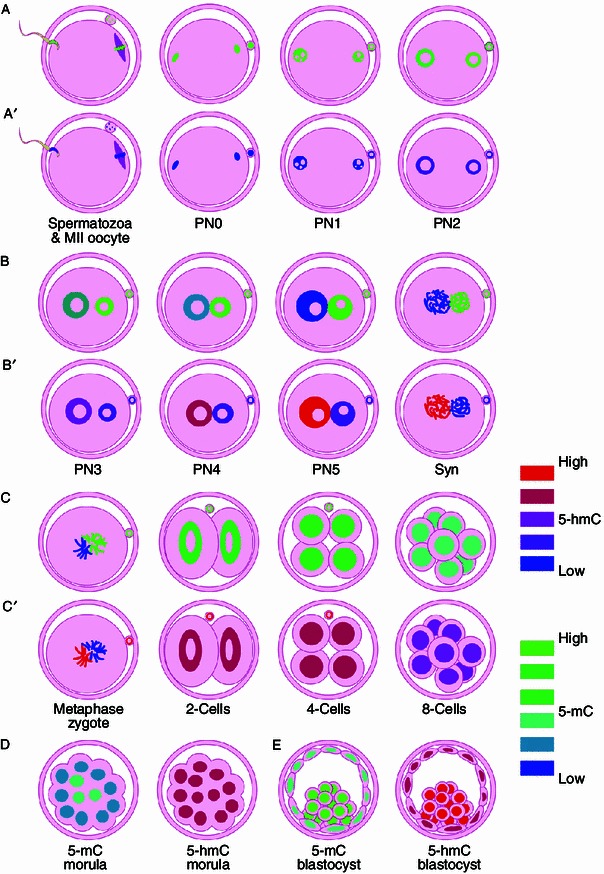
**DNA methylation dynamics in mouse preimplantation embryos**. (A, B, C, D left and E left), dynamics of 5-mCs in oocyte and preimplantation embryos; (A′, B′, C′, D right and E right), dynamics of 5-hmCs in oocyte and preimplantation embryos

After a spermatozoon fertilizes the oocyte, the protamines on the sperm chromatin are replaced by histones (van Meel and Pearson, 1979) before paternal pronucleus (PPN) and maternal pronucleus (MPN) formation. During subsequent early embryo development, the zygotic genome is firstly demethylated from the zygote to morula stage and the genomic DNA is then *de novo* methylated at the blastocyst stage (Ma et al., 2012; Santos et al., 2002; Smith et al., 2012) (Fig. 1). During demethylation of the zygotic genome, different demethylation mechanisms are utilized in maternal or paternal genomes. The paternal genome is actively demethylated (DNA replication independent and mediated by enzymes) whereas the maternal genome is mainly passively demethylated (diluted through DNA replication) (Inoue and Zhang, 2011; Rougier et al., 1998). For the active DNA demethylation, 5-methylcytosines (5-mCs) can be oxidized by the tet methylcytosine dioxygenase 3 (TET3) into 5-hydroxymethylcytosines (5-hmCs) (Iqbal et al., 2011; Ruzov et al., 2011). Although the maternal and paternal genomic CGIs undergo global DNA demethylation, by comparing the DNA methylation profiles of gametes with those of the blastocyst or inner cell mass (ICM) cells, evidence has shown that about half of the gamete-specific DNA methylation patterns is partially maintained during preimplantation embryo development (Borgel et al., 2010; Kobayashi et al., 2012; Smallwood et al., 2011).

Recent studies have revealed more details about the mechanisms underlying active DNA demethylation and the essential roles of zygotic genome reprogramming. Here we mainly review details of zygotic genome DNA demethylation and the molecules controlling active DNA demethylation during mammalian early embryo development.

## TET proteins mediate active dna demethylation

There are three TET proteins exist in mouse, Tet1, Tet2 and Tet3. All the TET proteins contain a cysteine-rich region and a double-stranded β-helix fold at their C-terminals taking functions as the catalytic domain. The Tet1 and Tet3 also contain a CXXC region, which can bind to the genomic CpG islands (Tan and Shi, 2012; Zhao and Chen, 2013). It was firstly discovered in Purkinje neurons and the brain that TET proteins mediate active DNA demethylation (Kriaucionis and Heintz, 2009; Tahiliani et al., 2009). TET proteins were shown to be partners of myeloid/lymphoid leukemia protein (MLL) protein, and oxidized 5-mC to 5-hmC depending on 2-oxoglutarate and Fe(II) and Vitamin C (Blaschke et al., 2013; Tahiliani et al., 2009). Further studies showed that the 5-hmCs could be further transformed into 5-formylcytosines (5-fCs) and 5-carboxylcytosines (5-caCs) by the TET proteins, and that the 5-fCs and 5-caCs could be replaced by cytosines through the base excision repair (BER) pathway (He et al., 2011; Ito et al., 2011; Song et al., 2013). The 5-hmC concentration in mammalian genomic nucleotide bases was less than 1% (Kriaucionis and Heintz, 2009), and traditional bisulfite sequencing methods could not distinguish 5-hmCs from 5-mCs (Huang et al., 2010), which increased the difficulty of investigating the biological functions of 5-hmCs. By using the biotin modified 5-hmC DNA fragments enrichment sequencing and immunofluorescence labeling, it was shown that the 5-hmCs were widely distributed in tissues and cultured cells (Ruzov et al., 2011; Song et al., 2011). The 5-hmC genome localization data of mouse embryonic stem cells (ESCs) showed that the 5-hmCs were mainly enriched at the gene body regions and formed peaks in the vicinity of transcription start sites (TSSs) (Williams et al., 2011). Data showed that 58% of 5-hmCs were identified at the gene body regions, and 6% at the promoter regions (Wu et al., 2011). Referring to the genome-wide ChIP-Seq data of chromatin code proteins, most 5-hmCs highly enriched promoters showed intensive histone H3 trimethyl Lys4 (H3K4me3) or H3K4me3/histone H3 trimethyl Lys27 (H3K27me3) signals, which indicated the 5-hmC highly enriched promoters were mostly corresponding to the active or the poised genes (Ficz et al., 2011; Pastor et al., 2011).

## Active DNA demethylation in early embryos

Active DNA demethylation in preimplantation embryos was firstly discovered in the mouse zygotic PPN (Mayer et al., 2000; Oswald et al., 2000). Immunofluorescence labeling results showed that the signals of PPN 5-mCs decreased firstly at the PN2 stage, and reached the lowest level at the PN4-PN5 stage. By subsequent dilution of the 5-mCs of paternal and maternal genomes by DNA replication-dependent passive DNA demethylation, the total genomic DNA 5-mCs reached the lowest level at the morula stage. At the blastocyst stage, the genomic DNA was remethylated (Santos et al., 2002).

The 5-hmC was firstly detected by immunofluorescence staining at both paternal and maternal pronuclei in the PN1 zygote. With zygote development, the 5-hmCs increased mainly in the PPN, which coincided with the decrease of PPN 5-mCs (Iqbal et al., 2011; Wang et al., 2014). Immunofluorescence labeling data showed that the 5-hmC also existed in the blastomeres of all embryo stages (Ruzov et al., 2011) (Fig. 1). Using chromosome spreading and 5-hmC labeling of blastomeres from the zygote to 8-cell embryo, Inoue and Zhang observed that 5-hmCs mainly localized at the paternal chromosomes and most 5-hmC positive genomic regions were demethylated in a DNA replication-dependent manner (Inoue and Zhang, 2011). However, very recent evidence showed that the 5-hmCs, the 5-fCs and the 5-caCs in early embryos were not just demethylated passively but that most of the oxidized derivatives of 5-mCs were demethylated actively (Wang et al., 2014).

## Active DNA demethylation-associated molecules in early embryos

**Figure 2:**
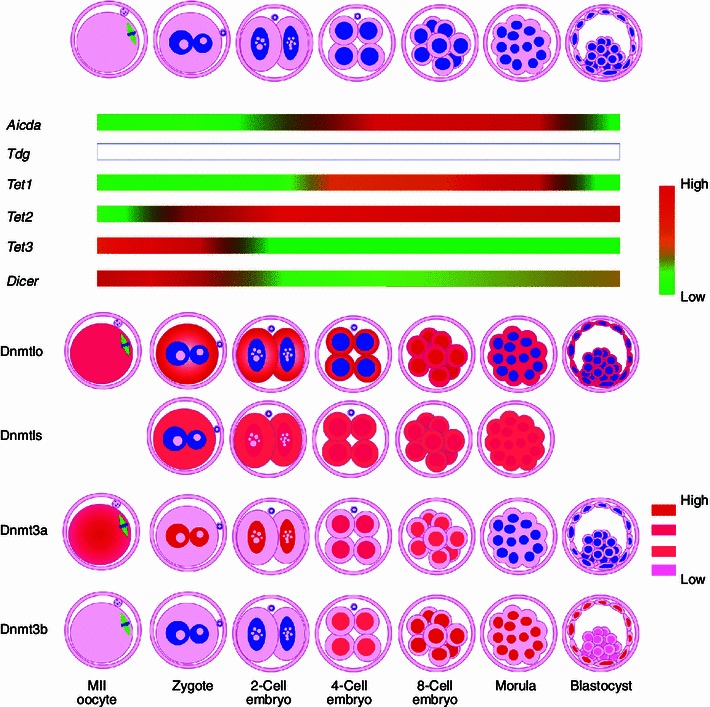
**Expression patterns of DNA demethylation-associated genes in mouse preimplantation embryos**. Aicda, (Kang et al., 2014); Tet1, Tet2 and Tet3, (Yu et al., 2013); Dicer1, (Murchison et al., 2007); Dnmt1o and Dnmt1 s, (Cirio et al., 2008; Hirasawa et al., 2008; Howell et al., 2001; Kurihara et al., 2008); Dnmt3a and Dnmt3b, (Hirasawa et al., 2008). Gene symbol: italic, mRNAs; bold regular, protein localization

The DNA methylation modification proteins could be classified into three categories: the DNA methyltransferase enzymes like DNA *de novo* methylation enzymes Dnmt3a, Dnmt3b and Dnmt3l, and the DNA methylation maintaining enzyme Dnmt1; the proteins that function in the active DNA demethylation pathways, and the unclassified factors such as the metabolite folic acid (Pufulete et al., 2005) and environment chemical biphenol A (Singh and Li, 2012). These molecules regulate the genomic DNA demethylation by coordination or by taking actions alone. However, the mechanisms by which they target the genomic-specific regions, regulate specific gene expression and participate in different developmental events are still unresolved. Here we summarize the functions of these proteins in preimplantation embryos. The expression levels and cellular localization of the main DNA demethylation associated proteins are displayed in Fig. 2.

### Roles of TET proteins in zygotic genome reprogramming

During early embryo development, all three TET proteins are expressed. QRT-PCR results showed that *Tet1* was highly expressed at the morula stage, *Tet2* mRNAs existed from the zygote stage through the blastocyst stage, and *Tet3* mRNAs mainly existed at the zygote stage. In mouse embryonic stem cells (ESCs), both Tet1 and Tet2 could bind to the *Nanog* gene promoter to catalyze 5-mC oxidation (Costa et al., 2013). Meanwhile, Gao et al. proved that *Tet1* could be used to replace the *Oct4* of Yamanaka factors (*Oct4*, *Sox2*, *Klf4* and *cMyc*, OSKM) to induce pluripotency in somatic cells (Gao et al., 2013). Recent data also showed that the OySyNyK [highly efficient modified factors of *Oct4*, *Sox2* and *Nanog*, which could induce the expression of Oct4-GFP of mouse embryonic fibroblast (MEF) as early as 24 h] increased *Tet1* expression at an early stage of somatic cell reprogramming (Zhu et al., 2014). A previous study showed that the expression of *Tet2* but not *Tet1* or *Tet3* was induced at the early stage of MEF reprogramming using OSKM and that the Tet2 protein could target the genomic sites of *Nanog* and *Esrrb* (Doege et al., 2012). For the human, evidence showed that TET2 but not TET1 or TET3 was essential for somatic cell reprogramming (Wang et al., 2013). By depletion of Tet1 and Tet2 in ESCs, it was shown that Tet1 and Tet2 indeed played distinct roles in DNA demethylation. Tet1 mainly targeted the transcription start sites whereas Tet2 mainly targeted the gene bodies (Huang et al., 2014). All the results from ESCs and iPSCs indicate that both Tet1 and Tet2 are associated with maintaining pluripotency of early embryonic cells.

Tet3 was mainly expressed in the zygote and located at the PPN (Nakamura et al., 2012). After knocking down Tet3 expression in mouse zygotes using siRNAs, the demethylation of PPN was diminished (Wossidlo et al., 2011), but the transcription activity showed no significant difference between Tet3 knock-down zygotes and normal zygotes (Inoue et al., 2012). In addition, transcript levels of transposable elements like *LINE1*, *ERVL* as well as the major satellite showed no obvious changes in zygotes or 2-cell embryos after Tet3 knock-down (Inoue et al., 2012), indicating that the global transcription of the zygote might not be affected by PPN active demethylation. In the Tet3-deficient zygotes, the activation of paternal *Oct4* gene was delayed, the reprogramming ability of zygotes was decreased, and the developmental failure of offspring increased (Gu et al., 2011), indicating that the epigenetic reprogramming induced by Tet3 at the zygote stage was important for further embryo development.

The Dppa3 (developmental pluripotency associated 3, also named PGC7 or Stella), is essential for the transition from the Non-Surrounded Nucleolus (NSN) type oocytes to the Surrounded Nucleolus (SN) type oocytes (Liu et al., 2012; Ma et al., 2013). When Dppa3 was depleted in the oocytes, the fertilized embryos could not develop beyond the 4-cell embryo stage (Bortvin et al., 2004). Evidence showed that the Dppa3 was mainly recruited to the MPN in zygote by the histone H3K9me2 to prevent the demethylation of MPN 5-mCs, when the zygotes were microinjected with the mRNAs of lysine (K)-specific demethylase 3A (Kdm3a, also known as Jhdm2a) which specially demethylates the H3K9me1 and H3K9me2, the levels of MPN H3K9me2 and Dppa3 decreased and the 5-hmCs level in MPN were increased (Nakamura et al., 2012). When Dppa3 was depleted, the 5-mCs of MPN could be oxidized to 5-hmCs by the TET proteins (Wossidlo et al., 2011). It was also shown that the DNA methylation levels of maternal imprinted genes like *Peg1*, *Peg3* and *Peg10*, paternal imprinted genes like *H19* and *Rasgrf1* and the transposable element *IAP* could not be maintained in the Dppa3-depleted zygote, whereas the DNA methylation levels of maternal imprinted genes *Snrpn* and *Peg5*, paternal imprinted genes *Meg3* and *LINE1* showed no obvious change when Dppa3 was depleted (Nakamura et al., 2007).

In addition to Dppa3, Tet3 protein localization is also controlled by the O-linked β-GlcNAc (O-GlcNAc) transferase (Ogt) and activation-induced cytidine deaminase (Aicda, also known as Aid). Tet3, but not Tet1 or Tet2, would be transferred to the cytoplasm when O-GlcNAcylated by Ogt (Zhang et al., 2014). When co-expressed with Aicda, the nuclear TET proteins would be translocated to the cytoplasm (Arioka et al., 2012).

### DNA methyltransferases

In preimplantation embryos, there are two isoforms of Dnmt1. The oocyte-specific Dnmt1o is mainly located at the cytoplasm of preimplantation embryonic blastomeres and enters the nucleus only at the 8-cell embryo stage (Howell et al., 2001). The oocyte-inherited somatic form Dnmt1s mainly existed at the zygote and 2-cell embryo stage, and at the 2-cell embryo stage the zygotic-originated Dnmt1s were expressed (Cirio et al., 2008). Dnmt1s located to the cytoplasm at the zygote stage and entered the nucleus in subsequent preimplantation embryo stages (Cirio et al., 2008) (Fig. 2). It was shown that maternal Dnmt1 was essential for the maintenance of genomic imprinting and the DNA methylation levels of transposable element *IAP* (Gaudet et al., 2004; Howell et al., 2001), *H19*, *Snrpn* and *Peg3* (Howell et al., 2001).

Both Dnmt3a and Dnmt3b are responsible for DNA *de novo* methylation, however, the target regions of Dnmt3a and Dnmt3b are not fully overlapping. The methylation of pericentric satellite DNA is controlled by Dnmt3b (Okano et al., 1999). The DNA methylation of genomic CGIs are mainly controlled by Dnmt3a with its parter Dnmt3l (Smallwood et al., 2011). The establishment of DNA methylation and its maintenance are regulated by the histone modifications (Kelsey and Feil, 2013). For example, the chromatin binding pattern of Dnmt3a showed negative correlation with that of the H3K4me3 (Smallwood et al., 2011). In addition to DNA *de novo* methylation activity, new evidence also showed that Dnmt3a and Dnmt3b could transform the 5-hmC to cytosines without the BER pathway (Chen et al., 2012), indicating the possible dual functions of Dnmt3a and Dnmt3b in preimplantation embryos.

### DNA damage repair associated proteins

During preimplantation embryo development, DNA double-strand breaks (DSB) occurred spontaneously at the zygotic PPN and blastomeres of 4-cell embryo, 8-cell embryo, morula and blastocyst (Ziegler-Birling et al., 2009). At the zygote stage, DNA DSBs coincided with the DNA replication of PPN and MPN. The DNA DSB marker, γH2A.X foci, appeared earlier and more abundant in PPN than in MPN (Derijck et al., 2008; Wossidlo et al., 2010). Although there was still no evidence proving the correlation between DNA DSBs and active DNA demethylation, chromosome spread results showed that the DNA DSB-induced sister chromatid exchange occurred in early embryos which might dilute the 5-mC on a single chromatid (Inoue and Zhang, 2011).

In addition to γH2A.X-marked DNA DSBs, proteins associated with BER and DNA single strand breaks (SSBs), such as the Parp1 [poly (ADP-ribose) polymerase family, member 1] and Xrcc1 (X-ray repair complementing defective repair in Chinese hamster cells 1), were also recruited to the paternal nucleus DNA (Hajkova et al., 2010). When the BER pathway was inhibited by apurinic/apyrimidinic endonuclease 1 (APE1) inhibitor (CRT0044876) or poly (ADP-ribose) polymerase family protein (PARP) inhibitor (3-aminobenzamide or ABT-888), the DNA demethylation of zygotic PPN became reduced (Hajkova et al., 2010).

In the BER pathway, the cytidine deaminase Aicda and thymine DNA glycosylase (Tdg) can recognize and excise the damaged bases. The Tdg could excise the 5-caC produced by TET proteins and the 5-hmUs which could be produced by deamination of 5-hmC mediated by Aicda (Ma et al., 2012). Tdg and Aicda play important roles in regulating DNA demethylation, however, both the Tdg and Aicda proteins are not detected in the mouse zygote (Hajkova et al., 2010). Tdg null embryos died at about embryonic day 10.5–12.5. Data from Tdg null MEFs showed that the DNA methylation levels increased in CpG islands. In addition, the histone modification H3K4me2 decreased whereas H3K27me3 and H3K9me3 increased in Tdg null MEFs (Cortazar et al., 2011).

The damage-specific DNA binding protein 1 (Ddb1) is the subunit of UV-DNA damaged DNA-binding protein complex and also the component of CUL4 complex which ubiquitinates the histones at UV-DNA damaged sites (Chen et al., 2001; Lan et al., 2012). When Ddb1 was depleted in the zygote, the Tet3-mediated 5-mC hydroxylation was blocked and the 5-mC level at PPN was maintained (Yu et al., 2013). All the above results indicate that the DNA repair pathway not only exerts functions directly on the active DNA demethylation but also maintains the progression of active DNA demethylation and repairs the active DNA demethylation-induced DNA damages.

### Other DNA demethylation-associated proteins or molecules

Dicer1 is a critical enzyme controlling the synthesis of miRNAs. In the Dicer1 null cells, the decrease of *miR-290* cluster miRNAs increased the expression of retinoblastoma-like 2 protein (Rbl2) which suppressed the expression of DNMTs (Benetti et al., 2008). RT-PCR data showed that the Dicer1 mRNAs mainly existed in fully grown oocytes and sharply decreased at the 2-cell embryo stage, indicating that Dicer1 exerted functions as a maternal effector. When conditionally knocking out of Dicer1 in growing oocytes, the Dicer1 null oocytes were mainly arrested at metaphase of the first meiosis (MI) stage (Murchison et al., 2007). Knocking out of Dicer1 induced the high expression of mouse transposon (*MT*) and *SINE* elements *B1* and *B2* (Murchison et al., 2007), which may be induced by abnormal DNA methylation pattern in Dicer1 null oocytes (Jeong and Lee, 2005). There is still no information about the effect of Dicer1 on early embryo DNA methylation dynamics, however, knocking down of Dicer1 could reduce the protein level of pluripotency factor Oct4 (Cui et al., 2007), indicating the pivotal roles of Dicer1 in preimplantation embryo development.

Other active DNA demethylation-associated factors include Elp3 (Okada et al., 2010), Gcm1 and Gcm2 (Hitoshi et al., 2011) and the mechanism details still need to be further analyzed.

## Active DNA demethylation is essential for embryo development

From the experiments of knockdown or knockout of genes like *Tet3*, it had been shown that the PPN DNA active demethylation was essential for mouse embryo development. As is well known, parthenogenetically activated oocyte-derived embryos mostly develop poorly after implantation and could not develop to term (Surani et al., 1984). However, Kono et al. had produced a full-term parthenogenetic mouse by activation of the MII oocyte which was composed of enucleated MII oocyte cytoplasm and the nucleus from another donor MII oocyte. The donor oocyte was produced by transferring the H19-depleted diplotene oocyte (from one day old mouse) nucleus into a fully grown oocyte, and if the nucleus was from the wild type, parthenogenetic embryos could only live for 14 days (Kono et al., 2004; Kono et al., 1996). In addition, the parthenogenetically derived ESCs could be used to produce live parthenote pups by tetraploid embryo complementation (Chen et al., 2009). These results may indicate that: firstly, paternal DNA demethylation was not essential for embryo development and the active DNA demethylation was critical for the development of mammalian placenta; and secondly, if the genomic imprinting was adaptive, embryos could develop to term without PPN active DNA methylation.

The 5-mC oxidation-mediated active DNA demethylation also occurred in somatic cell nuclear transfer (SCNT) mouse 1-cell embryos (Wossidlo et al., 2011). However, the quality of SCNT-produced embryos was obviously lower than that of *in vitro* fertilization (IVF) or intracytoplasmic sperm injection (ICSI)-induced zygotes, which indicated that genomic reprogramming of the somatic cell nucleus by the MII oocyte might not be complete (Thuan et al., 2010; Yang et al., 2007). Recent evidence showed that the nucleus of ESCs, fetal fibroblast or cumulus cells could be reprogrammed by the cytoplasm of interphase 2-cell embryo blastomeres after cell cycle synchronization, however, the interphase 2-cell embryo cytoplasm only could support the cell cycle synchronized ESC nuclear transfer embryo development to term (Kang et al., 2014). These results showed that the epigenetic code established during the preimplantation stage is essential for embryo development to term, in which active DNA demethylation is critically essential.

## Species-specific zygotic DNA demethylation

Immunofluorescence data showed that the DNA methylation dynamic patterns of different species are not conserved. For human, monkey, rat and mouse, the 5-mC signals were mostly lost in the PPNs whereas for the sheep, there was no obvious 5-mC signal lost in the PPN. The PPN 5-mC dynamic data of pig, cow, goat and rabbit is still not validated (Ma et al., 2012). Compared with the PPN 5-mC, the data of 5-hmC was not enough to analyze the conservation among mammalian species. Evidence showed that the 5-hmC signals mainly increased in PPNs of cow and rabbit (Wossidlo et al., 2011), whereas the 5-hmC signals existed in both the PPN and MPN of pig zygotes (Lee et al., 2014). TET3-induced DNA demethylation was critical for the maternal-zygote transition (MZT) and the expression level of NANOG in blastocysts of pig, which suggested that the 5-mCs hydroxydation was important for early porcine embryo development (Lee et al., 2014). Although the information about active DNA demethylation among mammals is still not sufficient, due to the conservation of TET proteins in these species, we suspect that the zygotic active DNA demethylation may be ubiquitous in higher mammals.

## Perspectives

**Figure 3:**
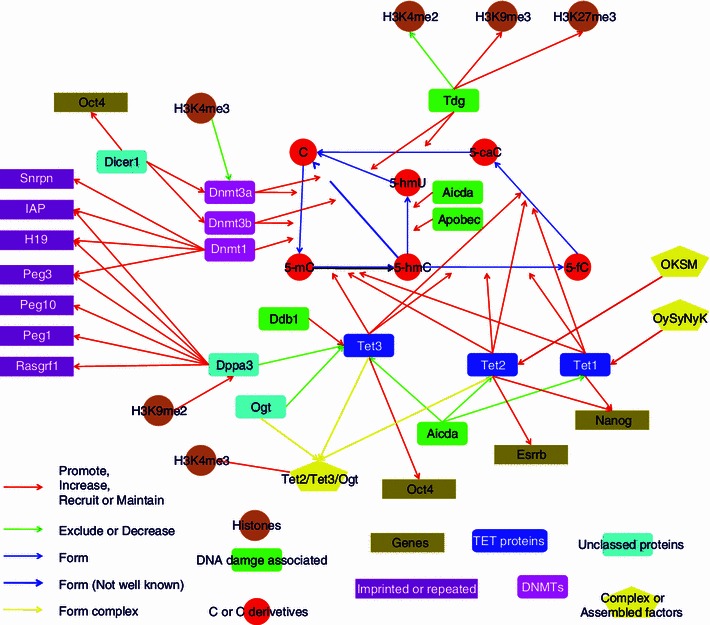
**Molecular network regulating DNA demethylation in preimplantation embryos**. The 5-mCs are mainly oxidated by TET proteins into 5-hmCs, 5-fCs or 5-caCs. The 5-hmCs could be targeted by Aicda, Apobec, Dnmt3a and Dnmt3b. The 5-caCs could be repaired into Cs (cytosines) by Tdg mediated base excision repair pathway. Most proteins participating directly in the DNA demethylation process could be taken as targets by other factors to regulate the DNA methylation pattern of specific genome regions, such as the localization of TET proteins could be affected by Aicda, Ogt and Dapp3, and the expression of *Tet* genes could be regulated by OSKM factors. In the preimplantation embryos, the DNA methylation imprinted genes and transposable elements *IAP* were mainly maintained by Dnmt1 and the Dapp3, however, the target sites of Dnmt1 and Dapp3 are different (see manuscript). In addition, the histone modifications and miRNAs can also affect or be affected by the active DNA demethylation process

The DNA demethylation in early embryos is complex and the DNA methylation patterns of CGIs, non CGI CpG sites, transposable elements, macro and minor satellites, and imprinted sequences are regulated by different proteins (Fig. 3). Although more and more details about active DNA demethylation have been discovered, many questions remain to be answered, including: Which distinct functions of the three TET proteins are critical for preimplantation embryo development? By which mechanisms is Dnmt1 specifially targeted to the imprinted regions and some repeat sequences and by which mechanisms are TET proteins targeted to specific 5-mCs? Whether or not the order of the gene promoter active DNA demethylation is important for embryo development? Are the DNA methylation pattern differences in different blastomeres affecting their developmental fates? What are the key DNA regions for SCNT embryos in reprogramming? How to change DNA methylation pattern of specific region artificially? And so on.

## Abbreviations

BER: base excision repair
CGI: CpG island
DSB: double-strand break
ICM: inner cell mass
ICR: imprinting control regions
MPN: maternal pronucleus
MZT: maternal-zygote transition
PPN: paternal pronucleus
SCNT: somatic cell nuclear transfer
SSB: single-strand break
